# How to live with a meningioma: experiences, symptoms, and challenges reported by patients

**DOI:** 10.1093/noajnl/vdaa086

**Published:** 2020-07-10

**Authors:** Farshad Nassiri, Suganth Suppiah, Justin Z Wang, Jetan H Badhiwala, Kyle Juraschka, Ying Meng, Romina Nejad, Karolyn Au, Nicole E Willmarth, Michael Cusimano, Gelareh Zadeh

**Affiliations:** 1 Division of Neurosurgery, Toronto Western Hospital, University of Toronto, Toronto, Canada; 2 MacFeeters Hamilton Center for Neuro-Oncology, Princess Margaret Cancer Center, Toronto, Canada; 3 Division of Neurosurgery, University of Alberta, Edmonton, Alberta; 4 American Brain Tumor Association, Chicago, USA; 5 Division of Neurosurgery, St. Michael’s Hospital, University of Toronto, Toronto, Canada

**Keywords:** fatigue, meningioma, patients, quality, survey

## Abstract

**Background:**

We aimed to explore gaps in the care of meningioma patients that could improve quality of care by better understanding symptoms experienced by patients at various stages of treatment, and afterwards.

**Methods:**

A novel 19-item self-administered questionnaire was provided for patients with meningiomas to complete by the American Brain Tumor Association (ABTA) over a 3-month period.

**Results:**

A total of 1852 unique respondents were included. Nearly one-third of all respondents felt they received insufficient information about meningiomas at initial diagnosis (*N* = 607, 32.9%) and 28.8% (*N* = 530) believed they received insufficient information about treatment options. In fact, 34.5% of respondents received the majority of their information from the internet and nonhealthcare professionals. The most common concerns after initial diagnosis were risks associated with surgery and/or treatment (36.5%) followed by how the tumor would impact daily life (25%) and the risk of tumor recurrence (12.4%). Respondents indicated that a list of resources available for patients with meningiomas (*N* = 597, 32.3%) would have been most beneficial in regards to their disease experience after their initial diagnosis. Moreover, we found that a substantial proportion of patients continued to report symptoms long after treatment, with fatigue being the most common compared to before treatment (38.2% vs. 57.7%, *χ*^2^ = 128, *P* < .001).

**Conclusions:**

Patients with meningiomas exhibit symptoms that continue well after treatment with fatigue and cognitive impairments as the most bothersome. Moreover, patients report key communication gaps that can be addressed to improve their disease experience and care.

**Key Points**

- Many meningioma patients desire additional information about diagnosis and treatment options.
- Pretreatment symptoms of meningiomas may persist long after treatment.
- Fatigue and cognitive decline may lower quality of life long after meningioma treatment.

**Importance of the Study**

This study reports on the largest available dataset for a patient/caregiver-reported survey on multiple dimensions of the meningioma disease experience. The results of this study confirm that patients with meningiomas exhibit symptoms that continue well after treatment with fatigue and cognitive impairments being the most concerning for them. Although patients should rely on healthcare professionals for medical information surrounding their diagnosis, the results of our study suggest that many resort to alternative sources such as the internet or nonhealthcare personnel, therefore identifying a key communication gap that can be addressed to improve the patient disease experience. Advocating for increased awareness on the short- and long-term impact of disease on patients allows practitioners to better understand areas for improvement and may provide rationale for increased funding or resources allocated to these needs.

Meningiomas are the most common primary brain tumor in adults. The rate of diagnosis of meningiomas is increasing for a variety of reasons including but not limited to the increased prevalence of neuroimaging and an increasingly aging population in which these tumours are more common.^1–4^ For tumors that cause mass effect or neurological symptoms, surgery is the mainstay of care with the goal of complete resection when possible.^2^ In most benign meningiomas, surgical cure can be achieved with low recurrence rates.^5^ Advances in surgical technique have improved prognosis and lowered complication rates considerably with most patients experiencing favorable outcomes with long-term progression-free and overall survival following treatment.^5–7^ While these objective measures of outcome have improved, the subjective impact of living with the diagnosis before and after treatment, as well as long-term requires more study.

The significant impairments in quality of life that both the diagnosis and treatment of brain tumors may portend is being increasingly recognized. Although most patients report improvements in health-related quality of life after treatment, there is growing evidence that suggests some patients may exhibit residual impairments long after completion of therapy.^8^ For example, patients with meningiomas report difficulties with physical, social, and emotional function, considerable neurocognitive impairments, particularly with memory, attention, and executive function that may significantly impair daily living.^9–12^ These reports highlight the need to place greater focus on patient-reported metrics, experiences, and subjective outcomes that are all key determinants of quality of life.

The purpose of the study was to elucidate the specific symptoms that are most bothersome to patients at various stages of treatment and clarify opportunities for improved patient–physician communication in order to improve the quality and delivery of patient-centred care. Here, we present the results of a self-administered survey of 1852 meningioma patients identified through the American Brain Tumor Association (ABTA).

## Methods

### Survey

We developed a novel 19-item self-administered online questionnaire with items tailored towards patients’ experiences with information seeking and their symptoms before and after treatment. The survey was refined by evaluating ease of use, content, and clinical sensibility and relevance by five different neurosurgeons. The survey was administered to patients identified to have meningiomas by the ABTA (Supplementary Table S1) and was made available through SurveyMonkey® to all constituents in the ABTA e-news mailing list (approximately 40 000 contacts) over a 3-month period. The contact list of the ABTA is formed by capturing contacts that reach out to the ABTA thorough its various programs and services including but not limited to patient and family meetings, careline, brochure requests, webinar attendance, volunteers, donors, past survey participants, peer-to-peer mentoring, fundraising events such as races, and e-news subscribers.

Additionally, targeted emails were sent to patients known to have a meningioma through the ABTA database. The survey was advertised through social media posts including Facebook®, Twitter®, LinkedIN® and promotion through the ABTA online support group and homepage.

### Statistical Analyses

Survey results, including multiple choice and free-text answers were reviewed by four authors (S.S., K.J., J.Z.W., and G.Z.). Respondents that did not indicate they themselves were diagnosed with a meningioma or responses from nonfirst-degree relatives/primary caregivers were excluded from analysis (*n* = 11). Pretreatment and posttreatment symptoms such as headache, weakness, seizures, fatigue, and impairments in coordination, vision, and hearing were recorded in a binary fashion (present/absent).

Data are reported as counts (and proportions), unless otherwise indicated. Categorical comparisons were performed using parametric Pearson Chi-square and nonparametric Mann–Whitney *U* testing when indicated. Patterns in free-text responses were explored using world clouds plotted using the *wordcloud* package in R which enables highlighting of the most frequently used keywords in a paragraph of texts with more frequent words represented as larger sized font. Statistical analysis was performed in R and *P* < .05 was considered to be statistically significant.

## Results

A total of 1852 unique fully completed questionnaires were included for analysis (Supplementary Table S1). Respondents were predominantly meningioma patients (83.3%), female (87.4%), and between the ages of 37 and 54 (90.2%). Only 16.7% of respondents were primary caregivers completing the survey on behalf of their first degree relative with a meningioma (Table 1).

**Table 1.** Baseline characteristics, treatment, and follow-up.

Respondents	# of patients (%)
Patients	1542 (83.3)
Primary caregivers/first-degree relative	310 (16.7)
Sex	
Male	233 (12.6)
Female	1619 (87.4)
Age	
≤19	29 (1.6)
20–34	118 (6.3)
35–44	361 (19.5)
45–54	608 (32.8)
55–64	481 (26.0)
65–74	207 (11.1)
>75	35 (1.9)
Undisclosed	14 (0.8)
Treatment offered at initial diagnosis	
Surgery/SRS	1213 (65.5)
As monotherapy	959 (79.1)
With radiotherapy^a^	440 (36.2)
Conservative management^b^	639 (34.5)
Reported treatment modality	*n* = 1552
Complete resection	964 (62.1)
Partial resection	362 (23.3)
Radiotherapy/radiosurgery	440 (28.3)
Chemotherapy	59 (3.8)
Time from symptoms to diagnosis	
≤6 months	864 (46.7)
7–12 months	245 (13.2)
13–24 months	186 (10.0)
>24 months	325 (17.5)
Unknown	212 (11.4)
Time from diagnosis to treatment/follow-up	
<3 months	1085 (58.6)
3–6 months	626 (33.8)
7 months–1 year	63 (3.4)
>1 year	78 (4.2)
Time from diagnosis to survey completion	
<3 months	88 (4.7)
7 months–1 year	152 (8.2)
> 1 year	406 (21.9)
> 3 years	360 (19.4)
> 5 years	732 (39.4)

SRS, stereotactic radiosurgery.

^a^Nonstereotactic radiotherapy as monotherapy or adjuvant therapy.

^b^With clinical and radiographic follow-up only.

Among all respondents, 1213 (65.5%) were offered treatment, including surgery, radiosurgery, or other forms of radiotherapy on initial diagnosis, while 639 (34.5%) were managed conservatively with serial radiographic and clinical follow-up (Table 1). Only 300 patients (16.2%) had not yet undergone treatment at the completion of the survey. Of those who underwent treatment, 79.1% had monotherapy with either surgery or a form of radiation. Most patients who underwent surgery reported a complete resection (62.1%). In addition, 440 (36.2%) respondents had radiation therapy either as monotherapy or adjuvant therapy. The majority of respondents (81.5%) had their diagnosis more than a year prior to completing the survey with 360 (19.4%) and 732 (39.4%) respondents having completed their treatment more than 3 and 5 years before the survey, respectively (Table 1).

### Sources of Information for Patients, Common Concerns, and Supports

**Figure 1.**
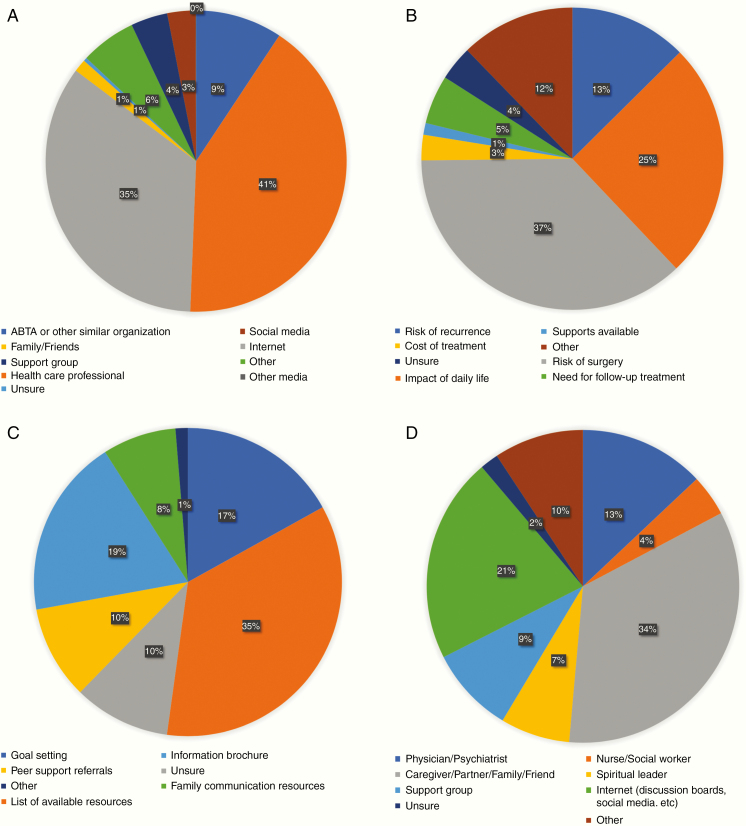
(A) Primary sources of information as reported by patients on their meningioma diagnosis and treatment options. (B) The most common concerns for patients reported after their initial meningioma diagnosis. (C) The most helpful source(s) of information as reported by patients following their meningioma diagnosis. (D) Sources of self-reported psychological and emotional support for meningioma patients.

**Figure 2.**
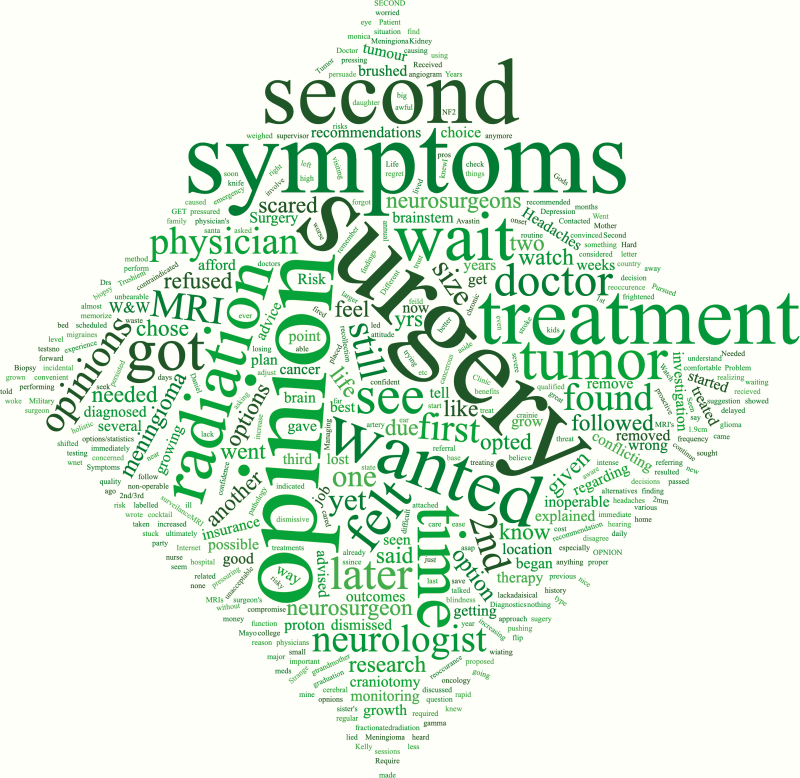
Word cloud for reasons given for not following physician instructions.

Nearly one-third (*n* = 607, 32.9%) of all respondents believed they received insufficient information about meningiomas at their initial diagnosis and 28.8% (*n* = 530) believed they received insufficient information about treatment options. The majority of respondents received most of their information on meningiomas from their healthcare providers (*n* = 761, 41.2%) (Figure 1A). Caregivers tended to report receiving information from their healthcare professional more frequently than patients (51% vs. 39%; Supplementary Figure S1). However, over one-third of all respondents (*n* = 638, 34.5%) received the majority of their information from the internet and other nonhealthcare sources (Figure 1A). There was no significant difference in reporting in patients under the age of 65 compared to those 65 and older (Supplementary Figure S5). Despite this, the overwhelming majority of respondents proceeded with their physician’s recommendations for treatment (91.9%) and most did not seek a second opinion (60.6%; Supplementary Table S2). The most common reason for not complying with their physician’s recommendations was a desire to explore other options or to seek a second, or third opinion or to avoid surgery altogether (Figure 2).

The most common concerns of respondents after their initial diagnosis were as followed: the risks involved with surgery/treatment (36.5%), how the tumor would impact their daily life (25%), and the risk of tumor recurrence (12.4%; Figure 1B). Reporting by patients themselves and their caregivers yielded largely congruent results (Supplementary Figure S2). There were no significant differences in the concerns reported by respondents age < 65 and ≥ 65 (Supplementary Figure S6).

Respondents ranked the following potential resources as the most helpful following their diagnosis: a dedicated list of trusted resources for meningioma patients (*N* = 597, 32.3%), brochures with detailed information on meningioma diagnosis and treatment (*N* = 319, 17.3%), and guidance regarding realistic goal setting (*N* = 287, 15.5%; Figure 1C). A higher proportion of patients reported goal setting as the most helpful priority compared to when caregivers completed the survey (20% vs. 12%; Supplementary Figure S3), but otherwise responses between the two parties were similar.

When asked regarding their primary sources of psychological or emotional support, the majority of respondents chose their caregiver, partner, family, or friend (*n* = 1037, 55.9%; Figure 1D). This was followed by internet resources such as patient message boards or social media (*n* = 646, 34.9%, a physician or psychiatrist (*n* = 394, 21.2%), or a support group (*n* = 271, 14.6%; Figure 1D). A higher proportion of patients reported using the internet or internet resource as a primary source of psychological support compared to when their caregivers completed the survey (23% vs. 14%; Supplementary Figure S4). More caregivers saw themselves, or family and friends as the primary source of emotional support for patients (38% vs. 33%). A slightly higher proportion of respondents age < 65 sought emotional support with their caregiver, family, or friends compared to respondents age ≥ 65, but there were no other meaningful differences (35% vs. 30%; Supplementary Figure S8).

### Pretreatment Self-Reported Symptoms

**Figure 3.**
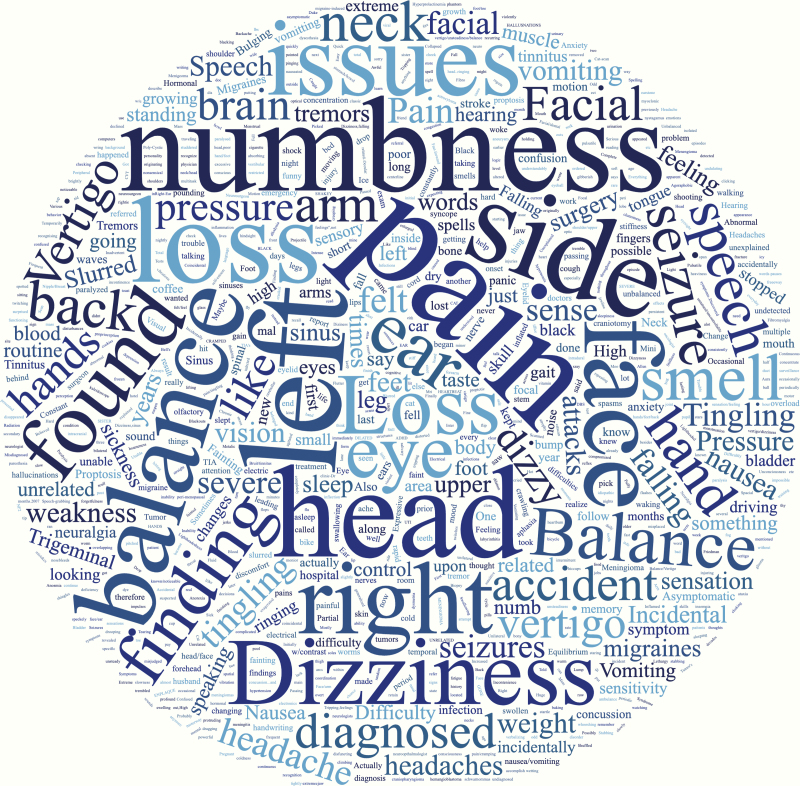
Word cloud of other subjective patient symptoms before diagnosis.

The most common symptoms at the time of diagnosis were headache (60.5%), fatigue (38.2%), trouble with vision (32.9%), cognitive impairment (27.1%), behavioral changes (22.1%), and motor weakness (20.6%; Table 2). Keywords for other subjective symptoms reported by patients prior to their diagnosis are represented in a word cloud (Figure 3). Overall, 864 (46.7%) respondents reported being diagnosed with a meningioma within 6 months of symptom onset (Table 1). Otherwise 245 (13.2%), 186 (10.0%), and 325 (17.5%) respondents reported being diagnosed 7–12 months, 13–24 months, and over 25 months after symptom onset, respectively, while an additional 212 (11.4%) respondents were unsure of timing. Interestingly, a higher proportion of caregivers reported fatigue (63.7%) and behavioral changes (33.7%) compared to patients themselves (39.5% fatigue; 20.0% behavioral changes; Supplementary Table S3). More respondents under the age of 65 reported fatigue (59.5%) as a presenting symptom compared to those over the age of 65 (35.8%), which may be reflective of employment status amongst other factors (Supplementary Table S4).

**Table 2.** Pre- and posttreatment symptoms at all time-points posttreatment.

Symptoms	Before diagnosis # of patients (%) *n* = 1852	After treatment # of patients (%) *n* = 1531
Headache	1119 (60.5)*	698 (45.6)*
Weakness in arm or leg	380 (20.6)	361 (23.6)
Poor coordination	211 (11.4)	244 (15.9)
Seizures	323 (17.4)	305 (19.9)
Cognitive deficit	502 (27.1)*	649 (42.3)*
Visual deficit	611 (32.9)	484 (31.6)
Hearing deficit	201 (10.8)	245 (16.0)
Behavioral changes	410 (22.1)	423 (27.6)
Fatigue	707 (38.2)*	884 (57.7)*
Other	642 (34.7)	N/A
Hormonal disorders	N/A	175 (11.4)

N/A, not applicable due to answer option not available in the survey.

*Indicates, statistical significance. Pearson’s Chi-square test, *P* < .01.

### Posttreatment Self-Reported Symptoms

**Figure 4.**
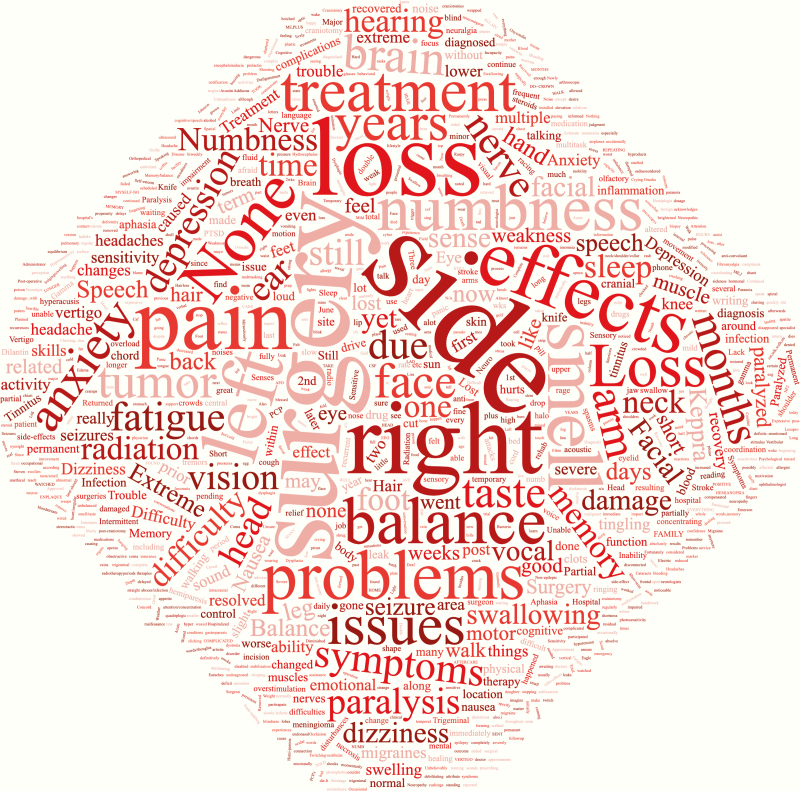
Word cloud of other subjective patient symptoms/side effects of treatment.

Persistent symptoms even after treatment remain substantial in patients with meningiomas (Table 2). In, fact the prevalence of prediagnosis symptoms remained relatively the same after treatment. There was a statistically significant decrease in the proportion of patients reporting headaches posttreatment (45.6% posttreatment vs. 60.5% pretreatment; *χ*^2^ = 74.1, *P* < .001). However, there was increased reporting of subjective cognitive deficits including memory, higher level thinking, etc. (42.3% posttreatment vs. 27.1% pretreatment; *χ*^2^ = 86.5, *P* < .001) and fatigue after treatment compared to before (57.7% vs. 38.2%; *χ*^2^ = 128, *P* < .001; Table 2, Figure 4). Interestingly, when responses were stratified into patient and caregiver respondents, caregivers noted a statistically significant decrease in fatigue after treatment (63.7% vs. 55.0%, *χ*^2^ = 4.1, *P* < .05) whereas patient respondents noted a significant increase, reflective of the total cohort (39.5% vs. 65.2%, *χ*^2^ = 184, *P* < .01; Supplementary Table S3).

Although there appears to be a statistically significant increase in the proportion of patients < age 65 with cognitive deficits after treatment (43.4% posttreatment vs. 27.9% pretreatment, *χ*^2^ = 75.5, *P* < .01), this increase is no longer significant when looking at patients ≥ 65 (Supplementary Table S4). Furthermore, in patients <65, there was a significant increase in reported hearing deficits posttreatment compared to before (16.3% vs. 10.8%, *χ*^2^ = 18.7, *P* < .01) whereas this is also not seen in patients ≥ 65. The higher proportion of presbycusis in more elderly population may contribute to this finding. Lastly, although a lower proportion of respondents ≥ 65 reported pretreatment fatigue (35.8%), there was a far more dramatic increase in the proportion of those reporting posttreatment fatigue (55.6%; *χ*^2^ = 16, *P* < .01) compared to respondents < age 65 (59.5% pretreatment vs. 64.5% posttreatment, *χ*^2^ = 7.6, *P* < .01; Supplementary Table S4).

Amongst all respondents 5-years out from their treatment, a substantial proportion of patients continued to report having headaches (38.7%), weakness (23.2%), behavioral changes (25.9%), and cognitive impairment (39.0%). In fact, proportion of patients reporting fatigue increased as time passed from the initial treatment. Only 31% of respondents within the first 3-months of treatment reported experiencing fatigue whereas 54% of respondents who were more than 5-years out reported fatigue as the most common symptom. When dichotomized, respondents who were more than 1-year out from treatment had a significantly higher frequency of fatigue compared to those who were treated within 1-year of completing the survey (*χ*^2^ = 18.1, *P* < .001).

## Discussion

Many central nervous system tumors such as gliomas and brain metastases result in symptoms and impairments in quality of life for a relatively short period of time due to early mortality.^13–16^ However, patients with meningiomas have a generally favorable prognosis and therefore are living with their disease and its stigmata for a prolonged period of time.^8,10,17^ Despite this, there are few studies on patient-based metrics, experience, and self-reported outcomes. In 2000, Kalkanis et al. reported on a telephone survey of 155 postoperative meningioma patients using 26 quality of life (QoL) questions on a Likert-scale based on the Functional Assessment of Patient Therapy-Brain study. They found that overall, a high proportion (over 80%) of patients reported satisfaction with their quality of life and independence in several domains. However, their study was limited by the number of respondents as well as follow-up time (mean follow-up time 33 months). Additionally, they focused only on postoperative patients, and did not ask any questions surrounding the diagnosis or pre/perioperative period.^18^ Benz et al. utilized the SF-36 to conduct a comprehensive 2017 study on health-related QoL in 1722 meningioma patients compared to 1622 control patients. They found that compared to healthy controls, postoperative meningioma patients had significant decreases in their QoL particularly in the domains of Role-Physical, Role-Emotional, Physical Functioning, and Social Functioning. However, their study was limited by the short interval between surgery and survey completion (median time 0.59 years), thereby limiting their assessment of long-term QoL, and as above, did not address pretreatment patient concerns.^19^ Our group conducted a prospective cross-sectional study on health related QoL in 291 postoperative meningioma patients in 2019 and found that a number of postoperative meningioma patients continued to experience fatigue, sleep impairment, emotional, and social functional impairment as far out as 120 months after treatment.^20^ Here, we present the results of a large, internationally administered survey that illustrates the patients’ experience and symptomology through various stages of the disease from the diagnosis to years after treatment, in the hopes of identifying opportunities to address unmet needs. To our knowledge, this is the largest survey of its kind on meningioma patients that attempts to address their entire clinical course.

Our study demonstrates that even 5-years after treatment, respondents continue to report experiencing fatigue (54.5%), cognitive impairment (39.0%), headaches (38.7%), weakness (23.2%), and behavioral changes (25.9%). These findings suggest that despite surgical cure, patients with meningiomas continue to have long-term effects from their initial tumor and may benefit from tailored neurocognitive and neuropsychiatric support in addition to physical rehabilitation.^11,20,21^ Furthermore, the fact that caregivers reported a higher prevalence of fatigue and behavioral changes than patients themselves may reflect their ability to perhaps more sensitively detect these changes in patients in order to motivate treatment earlier.

It is notable that the prevalence of self-reported fatigue at any time point was significantly higher after treatment compared to before (57.7% posttreatment vs. 38.2 pretreatment including all time points). This was particularly true for elderly patients, in which meningiomas are more commonly diagnosed. It is well known that fatigue is one of the most common adverse effects of cancer that can persist for years after treatment.^20,22–26^ A 2019 study by van der Linden et al. found that meningioma patients reported significantly higher levels of fatigue compared with the general population both before and 1-year after surgery with preoperative fatigue scores only weakly to moderately correlated with postoperative scores.^27^ Although meningiomas are not generally considered to be cancerous, the persistence of fatigue along with the perceived inability to return to baseline function is worthy of further investigation to better understand the underlying etiology. It may be that other pretreatment symptoms are more severe and mask the presence of fatigue, so it is more noticeable after those other symptoms subside. The fatigue experienced by cancer patients however is known to be more severe and debilitating than typical fatigue due to sleep deprivation or overexertion and is thought to be multifactorial with psychosocial, behavioral, and biological factors with some data suggesting it may be independent of the type of treatment a patient undergoes.^22,23,28^ Moreover, patients with cancer who have weaker social and emotional support systems report increased frequency and severity of fatigue.^24,29–31^ Other determinants of cancer-related fatigue include medical comorbidities, medications, physical symptoms, mental health, and physical deconditioning.^22,23,28^ Fatigue in cancer patients is correlated with decreased activity and physical deconditioning. Multiple studies have demonstrated that physical exercise during cancer treatment improves cancer related fatigue and helps to maintain quality of life.^26,30,32,33^ It is important to take lessons from the cancer literature to apply to meningiomas. Advocating for early mobilization, exercise, and behavioral modification after treatment of meningiomas may help alleviate some of the effects of meningioma related fatigue and improve overall quality-of-life in these patients. These interventions and their efficacy will need further investigation for this particular patient population and particularly for older patients with decreased physiological reserve compared to their younger counterparts.

This survey may also help leverage patient perspectives into opportunities for improving care in meningiomas. Perhaps the most notable and addressable need that emerged was the patients’ desire for more information on diagnosis and treatment. Approximately one-third of survey respondents reported receiving what they felt was inadequate information about their disease at the time of diagnosis and a similar proportion felt they had received inadequate information on treatment. Furthermore, nearly a third of respondents reported having learned the majority of their disease information from the internet instead of from their healthcare providers. Despite this, patients still have a high level of confidence in their physicians as evidenced by their overwhelming compliance regarding treatment recommendations. However, about one-third of patients sought second opinions. These results reinforce the need for physicians and other healthcare providers to optimize communication strategies and develop adjuncts to effectively counsel patients about their diagnosis and treatment plan. Interestingly, the respondents in our study reported that even simple interventions such as providing a list of locally and nationally available resources for patients with meningiomas and/or generic brochures with information regarding meningiomas would have been extremely helpful. Raising awareness on how to access existing, reliable information and focusing on preparing patient-forward communication will play an increasingly large part in the role of a physician.

The most notable limitations of this study relate to the use of a survey and its associated recall bias whereby respondents may be more or less likely to recall certain information on exposure depending on their outcome status or vice versa.^34,35^ For example, patients who are further removed from their treatment may have more difficulty recalling pretreatment symptoms with accuracy.^34^ Additionally, more specific descriptors than “cognitive impairment” or “behavioral change” such as “memory loss,” “poorer work performance,” “poorer school performance,” etc. may also facilitate more accurate recall and reporting.^36^ The severity of symptoms may also affect the accuracy of recall which in many cases was not evaluated in this survey as it was intended to only report whether patients did or did not experience certain symptoms.^37^ Also, as an online survey provided by a brain tumor support group, there are implicit biases in who preferentially responds. For example, those with ready access to the internet or those who are more technologically savvy are more likely to participate, and those more engaged with the organization prior to survey distribution which may include a higher proportion of symptomatic meningioma patients, may be more likely to complete the survey. Furthermore, based on the wording of our survey, though patients may report certain symptoms more than 5-years after their treatment, this does not always imply these deficits were persistent and longstanding since their treatment, but may have in fact newly arisen anytime from their treatment to completion of the survey, whether it be due to recurrence, salvage treatment, etc. However, our survey results do not provide granular enough data to explore this in more detail. Lastly, there was a relatively lower proportion of elderly patients aged 65 or older (<14% of total respondents) who responded to this survey which may have been due to its online/electronic nature which is certainly suboptimal when considering that meningiomas are more common in patients over the age of 50. This sampling bias may be ameliorated in the future with the use of telephone surveys to supplement the online distribution.

## Conclusion

This study presents the largest available dataset on a patient/caregiver-reported survey on multiple dimensions of the meningioma disease experience. The results of this study confirm that patients with meningiomas exhibit symptoms that continue well after treatment with fatigue and cognitive impairments becoming more prominent posttreatment. Although patients rely on their healthcare professionals for medical information, the results of our study bring to light key communication gaps that can be bridged to improve patient care. Advocating for increased awareness of the impact of this disease on patients, both in the short- and long-term, and better understanding of the resources, and support patients may need will contribute to overall greater care for those with meningiomas before and after treatment.

## Supplementary Material

vdaa086_suppl_Supplementary_Material

## Notes

**Authorship Statement**. Conception and design: N.W. and G.Z.; collection and assembly of data: F.N., S.S., J.Z.W., K.J., Y.M., R.N., K.A., M.D.C., and G.Z.; data analysis and interpretation: F.N., S.S., J.Z.W., K.J., Y.M., R.N., K.A., M.D.C., and G.Z.; manuscript writing: F.N., S.S., J.Z.W., and G.Z.; final approval of manuscript: all authors.
