# Supplementary material for: How to live with a meningioma: experiences, symptoms, and challenges reported by patients
Source: Neurooncol Adv. 2020 Jul 10;2(1):vdaa086. doi: 10.1093/noajnl/vdaa086 (PMC7415257; doi:10.1093/noajnl/vdaa086)
Supplement: vdaa086_suppl_Supplementary_Material [file vdaa086_suppl_supplementary_material.docx]

**Supplementary Figure and Captions:**

**Supplementary Figure 1.** Primary sources of information as reported by patients and caregivers on their meningioma diagnosis and treatment options.

**
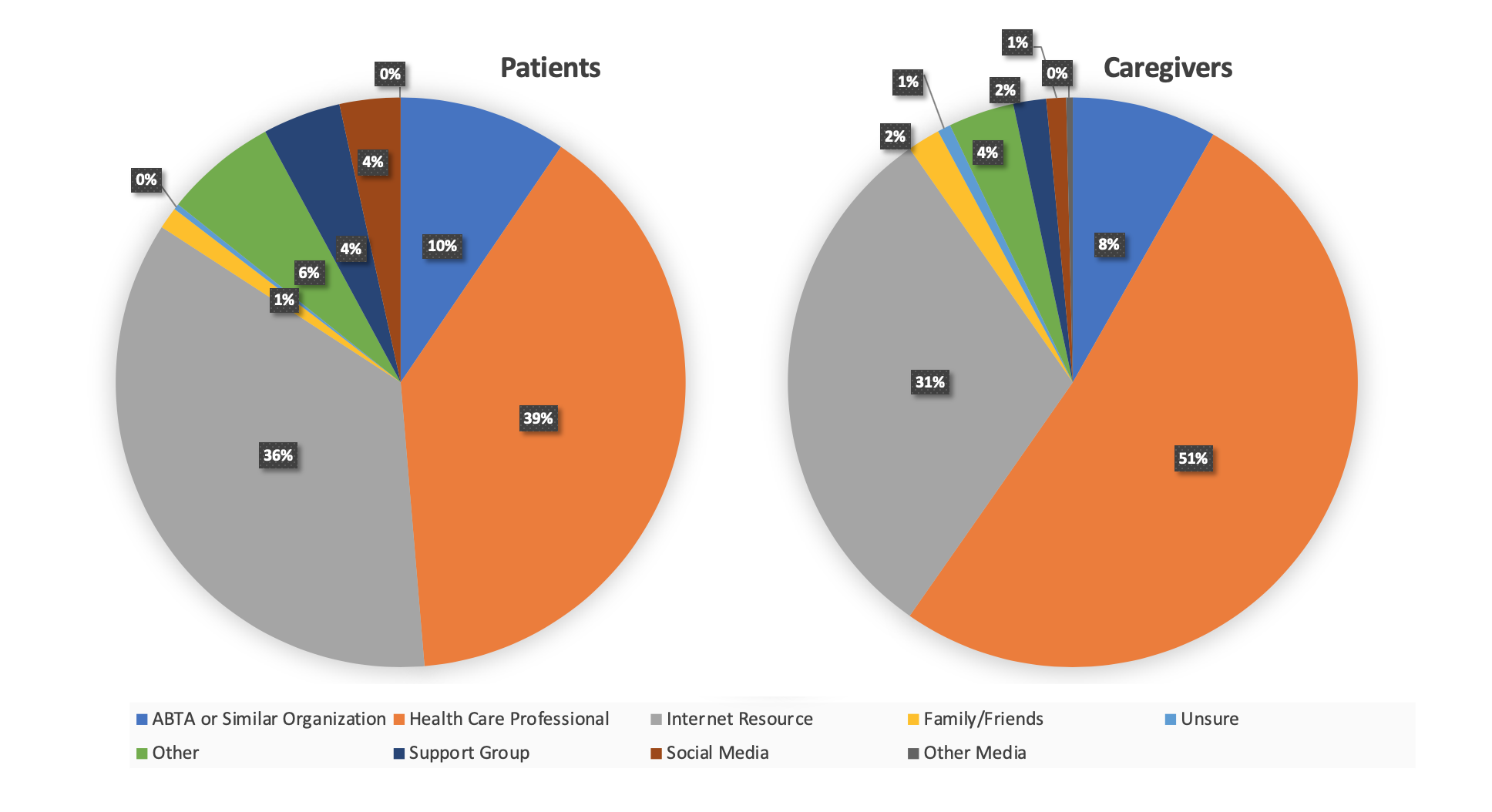
**

**Supplementary Figure 2.** The most common concerns for patients reported after their initial meningioma diagnosis as reported by patients and caregivers.

**
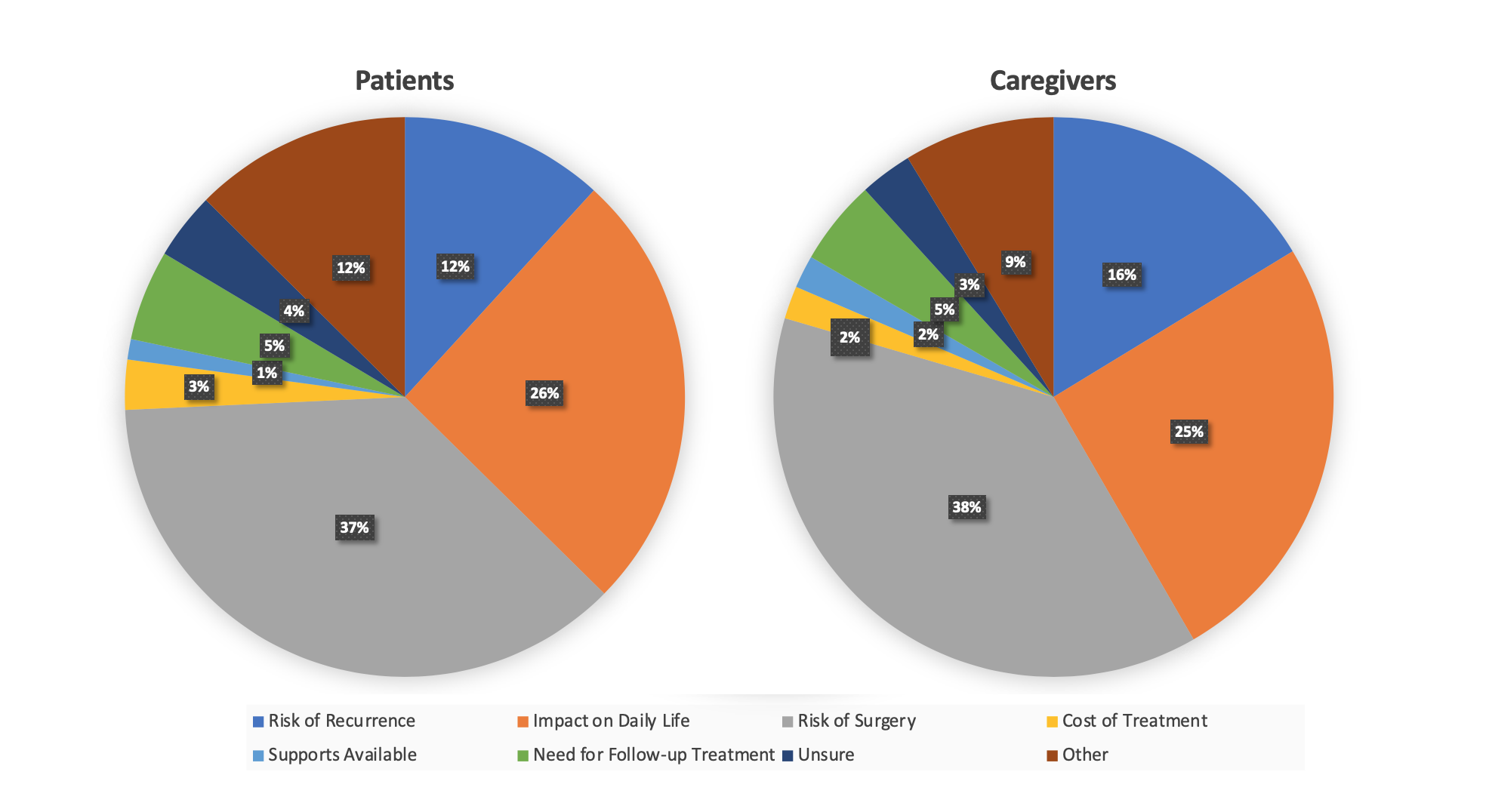
**

**Supplementary Figure 3.** The most helpful source(s) of information as reported by patients and caregivers following their meningioma diagnosis.

**
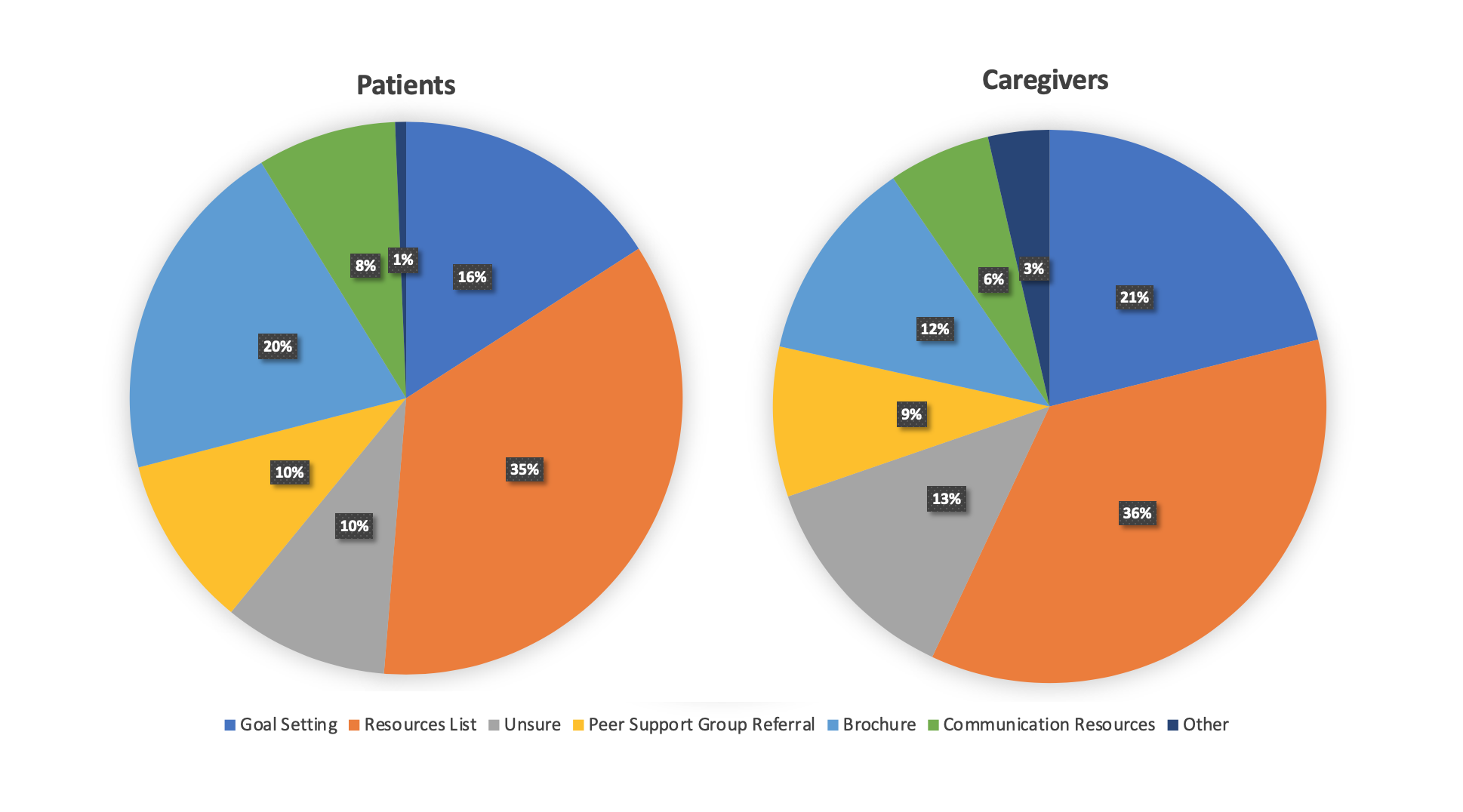
**

**Supplementary Figure 4.** Sources of psychological and emotional support as reported by patients and their caregivers

**
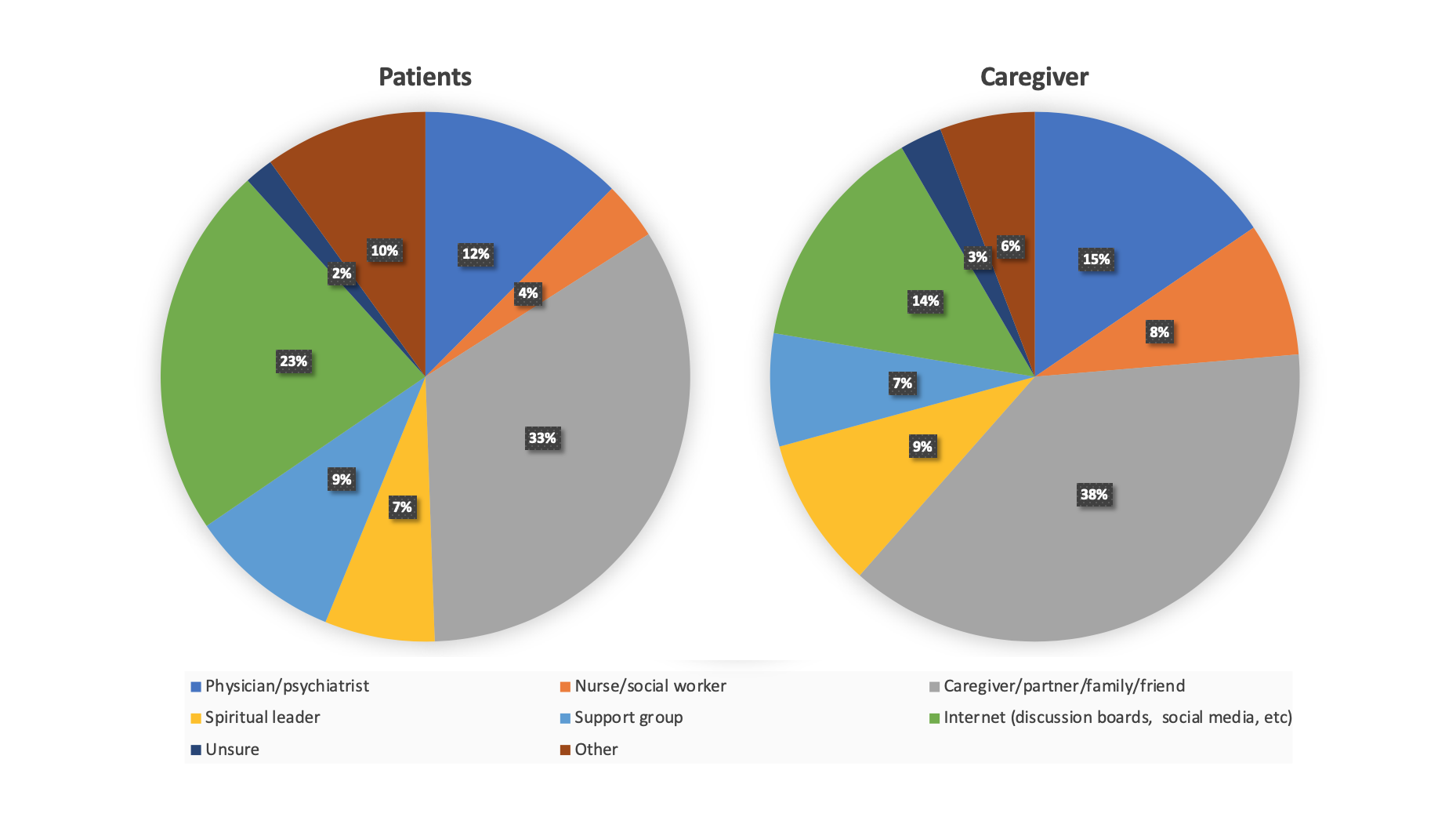
**

**Supplementary Figure 5.** Primary sources of information as reported by patients age <65 and ≥ 65 on their meningioma diagnosis and treatment options

**
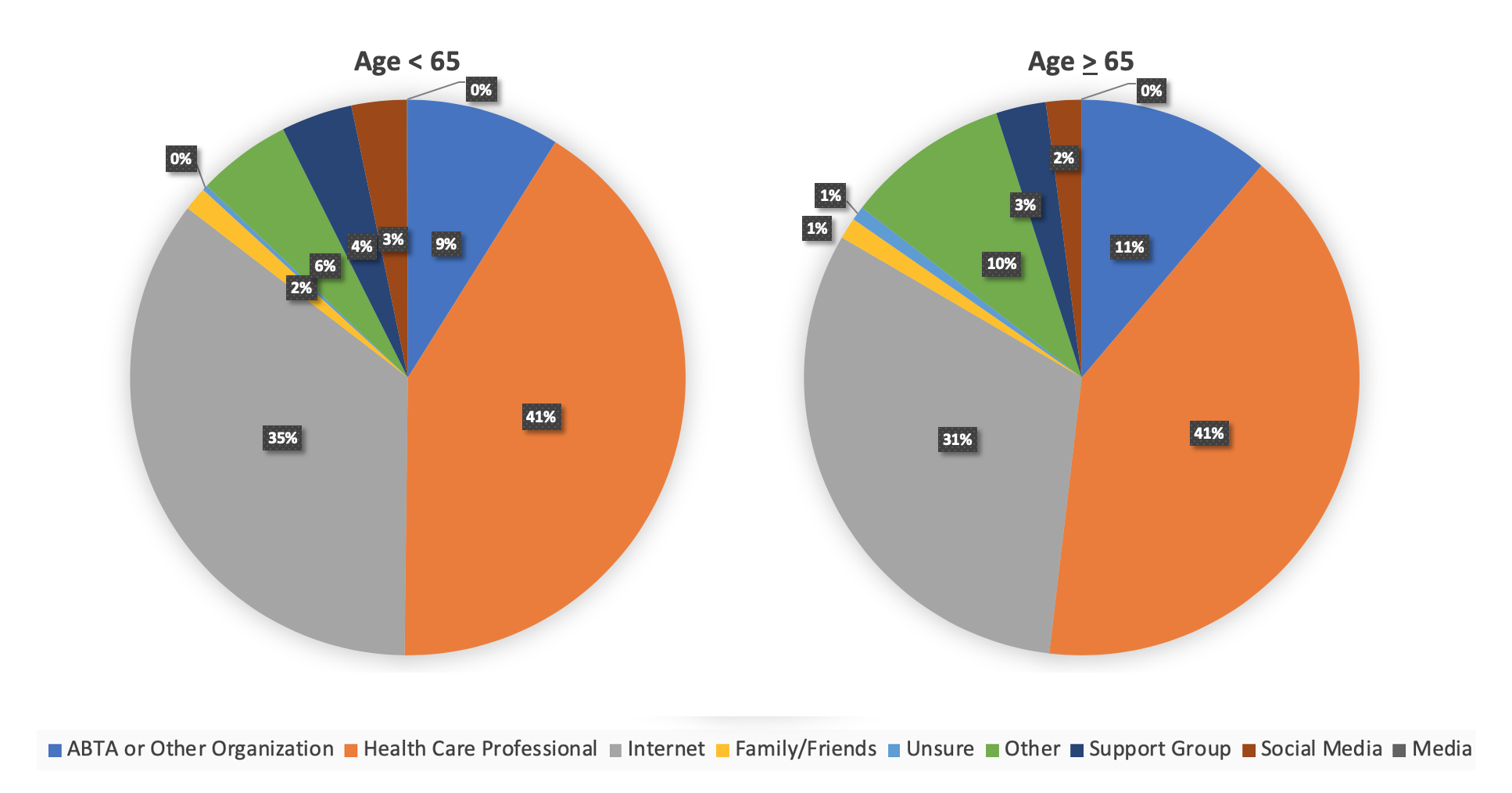
**

**Supplementary Figure 6.** The most common concerns for patients age <65 and ≥ 65 reported after their initial meningioma diagnosis.


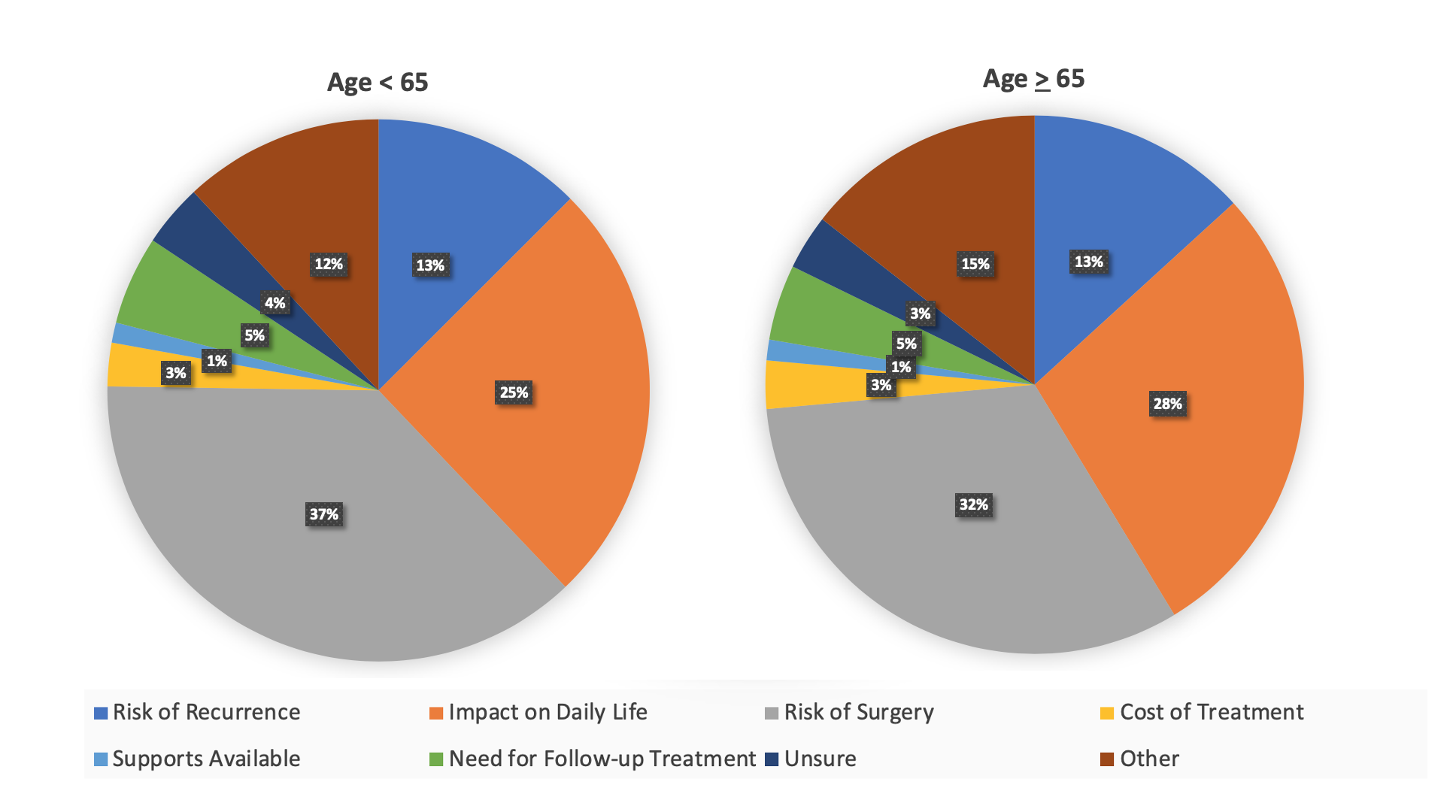


**Supplementary Figure 7.** The most helpful source(s) of information as reported by patients patients age <65 and ≥ 65 following their meningioma diagnosis.

**
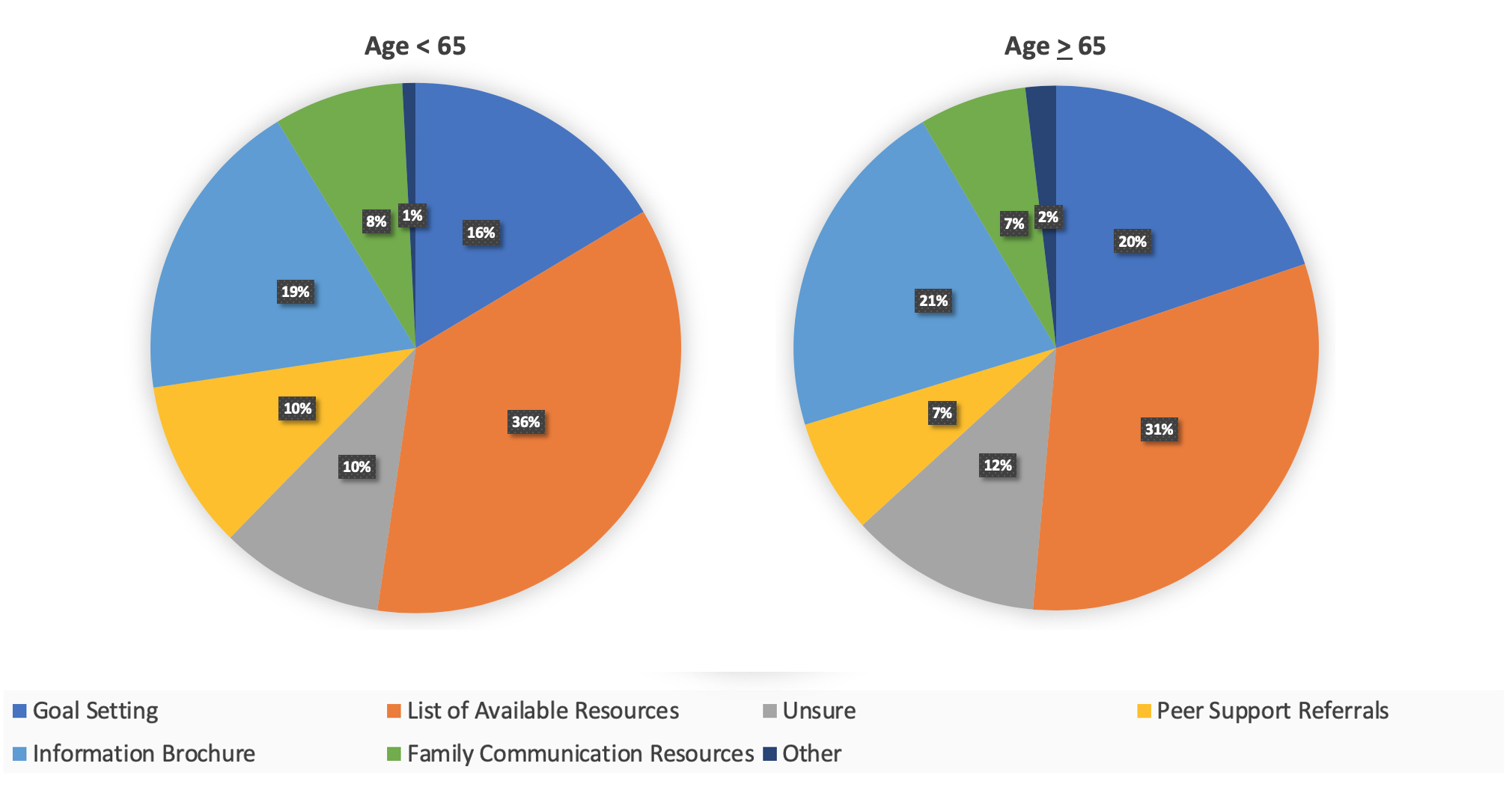
**

**Supplementary Figure 8.** Sources of psychological and emotional support reported by patients aged < 65 and ≥ 65.

**
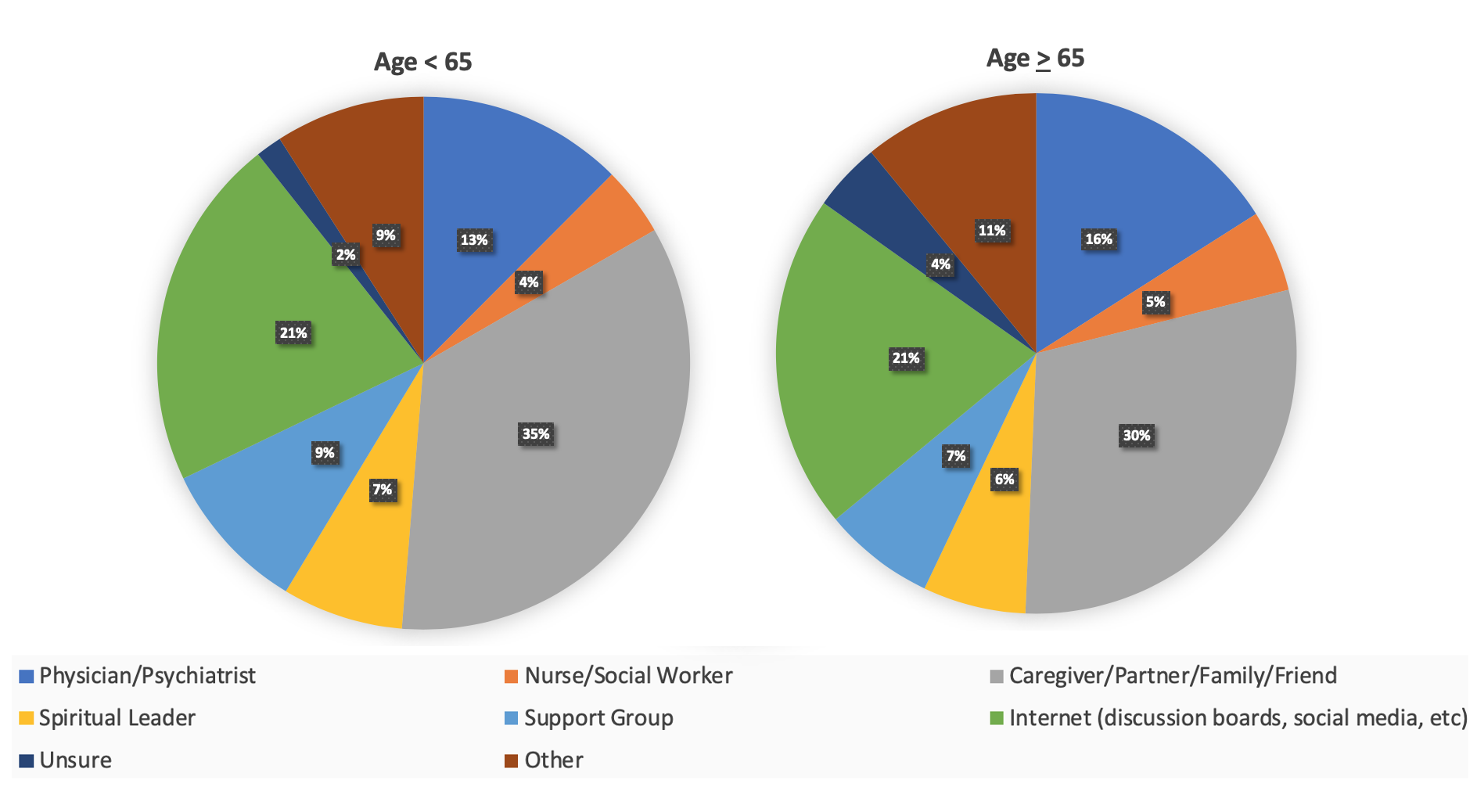
**

**Supplementary Tables:**

**Supplementary Table 1.** ABTA Survey Items excluding basic patient identifiers/demographics

| Question | Response Choices |
| --- | --- |
| I am a… | 1. Patient 2. Caregiver of someone diagnosed with a meningioma 3. Other: specify |
| 1. What were your/the patient’s symptoms before diagnosis? Check all that apply | 1. Headache 2. Weakness in arm or leg 3. Trouble coordinating arms or legs 4. Seizures 5. Cognitive function (memory, thinking, etc.) 6. Trouble with vision 7. Difficulty with vision 8. Behavioral changes 9. Fatigue 10. Unsure 11. Other (please specify) |
| 1. How long did it take from the time symptoms were first experienced until you/the patient received a brain tumor diagnosis? | 1. 0-6 months 2. 7-12 months 3. 13-24 months 4. Over 25 months |
| 1. How long has it been since the diagnosis? | 1. Less than 3 months 2. 3 to 6 months 3. 7 months to 1 year 4. More than 1 year 5. More than 3 years 6. More than 5 years |
| 1. What was your/the patient's primary concern after diagnosis? (select one) | 1. How the tumor would impact daily life 2. What the risks were for tumor recurrence 3. Risks involved with surgery 4. The cost of treatment 5. Need for follow-up care or additional treatments 6. The availability of support services 7. Other (please specify): 8. Unsure |
| 1. Did you receive information about meningiomas? | 1. Yes 2. No 3. Unsure |
| - Did you receive information about treatment options? | 1. Yes 2. No 3. Unsure |
| 1. What additional information would you have found the most beneficial after diagnosis? (select one) | 1. Brochure with detailed information on meningiomas 2. A list of currently available resources specific to meningioma 3. Realistic goal setting 4. Referral to peer support group 5. Information on how to talk to others about diagnosis 6. Other (please specify): 7. Unsure |
| 1. Where did you/the patient turn to for psychological/emotional support? (check all that apply) | 1. Physician/Psychiatrist 2. Nurse/social worker 3. Caregiver/partner/family/friend 4. Spiritual leader 5. Support group 6. Internet, including discussion boards and social media 7. Unsure 8. Other (please specify): |
| 1. Did you have any interest in connecting with other meningioma patients or caregivers, and if so how? (check all that apply) | 1. No 2. Yes, through the hospital/physician/nurse/social worker 3. Yes, through a support group 4. Yes, through a brain tumor or other advocacy organization, such as the American Brain Tumor Association 5. Yes, through the internet (discussion groups, blogs, etc.) 6. Yes, through social media 7. Unsure 8. Other (please specify): |
| 1. After diagnosis, did the physician recommend "waiting and seeing"/monitoring or proceeding into treatment? | 1. “Wait and see”/monitor 2. Proceed into treatment 3. Unsure |
| 1. Did you/the patient follow the physician's recommendation? | 1. Yes 2. No because… (please specify): |
| 1. Did you get a second opinion? | 1. Yes 2. No |
| 1. If you/the patient proceeded into treatment, how long did it take between diagnosis and starting treatment? | 1. Less than 3 months 2. 3-6 months 3. 7-12 months 4. More than 1 year 5. More than 5 years 6. Not applicable (did not move into treatment) |
| 1. If you/the patient proceeded into treatment, how was the brain tumor treated? (check all that apply) | 1. Surgery: complete resection (complete removal of tumor) 2. Surgery: partial resection (partial removal of tumor) 3. Radiation therapy/Radiosurgery 4. Chemotherapy 5. Unsure 6. Not applicable (I was not moved into treatment) 7. Other (please specify): |
| 1. Did you receive any information about potential treatment side-effects? | 1. Yes 2. No 3. Unsure |
| 1. After treatment, what side-effects did you/the patient experience? (check all that apply) | 1. Headache 2. Weakness in arm or leg 3. Trouble coordinating arms or legs 4. Seizures 5. Cognitive function (memory, thinking, etc.) 6. Trouble with vision 7. Difficulty with hearing 8. Behavioral changes 9. Fatigue 10. Hormonal disorders 11. Not applicable (I did not proceed to treatment) 12. Other (please specify) |
| 1. After treatment, has the meningioma recurred? | 1. Yes 2. No 3. Unsure |
| 1. If the meningioma recurred, how long was it after initial treatment? | Free text |
| 1. Did you/the patient participate in a clinical trial? | 1. Yes 2. No 3. Unsure |
| - If you/the patient did participate in a clinical trial, what contributed to making that decision? (check all that apply) | 1. Physician recommendation 2. Family/friend support 3. Feedback from someone who has participated in a clinical trial 4. Found out more information about clinical trials online or from another source 5. Not applicable (did not participate in a clinical trial) 6. Unsure if the patient participated in a clinical trial |
| - If you/the patient did not participate in a clinical trial, why not? (check all that apply) | 1. The doctor did not provide that as an option 2. Not aware of available clinical trials 3. Not eligible for any clinical trials 4. Not interested in participating in a clinical trial 5. There were no clinical trials close to me/the patient 6. Financial concerns 7. Unsure about the risks and benefits 8. Not applicable (did participate in a trial) 9. Unsure if the patient participated in a clinical trial 10. Other (please specify): |
| 1. How was your/the patient's career/school impacted by the brain tumor diagnosis? | 1. An extreme amount 2. A moderate amount 3. A significant amount 4. A little bit 5. Not at all 6. No applicable/unsure |

**Supplementary Table 2**. Other results of various survey questions

| **Receive general information regarding meningiomas?** | | **# of patients (%)** |
| --- | --- | --- |
|  | Yes | 1146 (61.9) |
|  | No | 608 (32.8) |
|  | Unsure | 98 (5.3) |
| **Receive treatment information regarding meningiomas?** | |  |
|  | Yes | 1237 (66.8) |
|  | No | 530 (28.6) |
|  | Unsure | 85 (4.6) |
| **Interest in connecting with other meningioma patients/caregivers** | |  |
|  | Yes |  |
|  | via healthcare | 213 (11.5) |
|  | via support group | 477 (25.8) |
|  | via brain tumor/advocacy organization | 474 (25.6) |
|  | via internet | 728 (39.3) |
|  | via social media | 488 (26.3) |
|  | Unsure | 155 (8.3) |
|  | Other | 145 (7.8) |
|  | No interest | 335 (18.1) |
| **Source of psychological or emotional support** | |  |
|  | Physician/psychiatrist | 394 (21.2) |
|  | Nurse/social worker | 130 (7.0) |
|  | Caregiver/partner/family/friend | 1037 (55.9) |
|  | Spiritual leader | 218 (11.8) |
|  | Support Group | 271 (14.6) |
|  | Internet e.g. discussion boards, social media | 646 (34.9) |
|  | Unsure | 58 (3.1) |
|  | Other | 282 (15.2) |
| **Treatment modality proposed after initial diagnosis** | |  |
|  | Monitor | 604 (32.6) |
|  | Proceed with treatment | 1207 (65.2) |
|  | Unsure | 41 (2.2) |
| **Did patient follow physician’s recommendation?** | |  |
|  | Yes | 1702 (91.9) |
|  | No | 117 (6.3) |
|  | Unsure | 33 (1.8) |
| **Was a second opinion sought?** | |  |
|  | Yes | 705 (38.1) |
|  | No | 1123 (60.6) |
|  | Unsure | 24 (1.3) |
| **Tumor recurrence after treatment?** | |  |
|  | Yes | 340 (21.1) |
|  | No | 936 (50.5) |
|  | Unsure | 338 (18.4) |
| **Participation in clinical trial** | |  |
|  | Yes | 70 (3.8) |
|  | No | 1658 (89.4) |
|  | Unsure | 35 (1.8) |
| **Decision to participate in clinical trial influenced by:** | | **n = 70** |
|  | Physician | 64 (91.4) |
|  | Family/friend | 17 (24.3) |
|  | Other trial participant(s) | 7 (10) |
|  | Online information | 14 (20) |
|  | Unsure | 40 (57.1) |
| **Decision to NOT participate in clinical trial due to:** | | **n = 1658** |
|  | Not provided as an option | 1084 (65.4) |
|  | Unaware of current trials | 668 (40.2) |
|  | Not eligible | 74 (4.5) |
|  | No interest | 60 (3.6) |
|  | Geographical limitations | 78 (4.7) |
|  | Financial concerns | 35 (2.1) |
|  | Uncertain of risks/benefits | 65 (3.9) |
|  | Unsure | 22 (1.3) |
| **Impact of disease on career/schooling** | |  |
|  | Extreme | 450 (24.3) |
|  | Significant | 364 (19.7) |
|  | Moderate | 327 (17.6) |
|  | A little | 312 (16.8) |
|  | Not at all | 194 (10.4) |
|  | N/A or Unsure | 205 (11.2) |

**Supplementary Table 3**. Patient symptoms as reported by patients and caregivers of patients

|  | **Patients** | | **Caregivers** | |
| --- | --- | --- | --- | --- |
| **Symptoms** | **Before Diagnosis**  **# of patients (%)**  **n = 1542** | **After Treatment**  **# of patients (%)**  **n = 1253** | **Before Diagnosis**  **# of patients (%)**  **n = 270** | **After Treatment**  **# of patients (%)**  **n = 249** |
| Headache | **955 (61.9)*** | **584 (46.6)*** | **142 (52.6)*** | **104 (41.7)*** |
| Weakness in arm or leg | 300 (19.5) | 276 (22.0) | 68 (25.1) | 76 (30.5) |
| Poor coordination | 161 (10.4) | 183 (14.6) | 40 (14.8) | 54 (21.6) |
| Seizures | 246 (15.9) | 232 (18.5) | 62 (22.9) | 61 (24.5) |
| Cognitive deficit | **404 (26.2)*** | **516 (41.2)*** | **89 (32.9)*** | **118 (47.3)*** |
| Visual deficit | 499 (32.3) | 400 (31.9) | 95 (35.1) | 77 (30.9) |
| Hearing deficit | 173 (11.2) | 205 (16.4) | 25 (9.3) | 39 (15.6) |
| Behavioral Changes | 309 (20.0) | 312 (24.9) | 91 (33.7) | 100 (40.2) |
| Fatigue | **609 (39.5)*** | **818 (65.2)*** | **172 (63.7)*** | **137 (55.0)*** |

*Pearson’s Chi-square test with Yates correction, p < 0.01

**Supplementary Table 4.** Pre- and post-treatment patient symptoms in patients under the age of 65 and those older than and equal to age 65.

|  | **Age < 65** | | **Age > 65** | |
| --- | --- | --- | --- | --- |
| **Symptoms** | **Before Diagnosis**  **# of patients (%)**  **n = 1597** | **After Treatment**  **# of patients (%)**  **n = 1333** | **Before Diagnosis**  **# of patients (%)**  **n = 243** | **After Treatment**  **# of patients (%)**  **n = 187** |
| Headache | **976 (61.1)*** | **637 (47.8)*** | **149 (61.3)*** | **60 (32.1)*** |
| Weakness in arm or leg | 336 (21.0) | 312 (23.4) | 48 (19.7) | 46 (24.6) |
| Poor coordination | 184 (11.5) | 209 (15.7) | 33 (13.6) | 33 (17.6) |
| Seizures | 269 (16.8) | 262 (19.7) | 44 (18.1) | 40 (21.4) |
| Cognitive deficit | **447 (27.9)*** | **579 (43.4)*** | 59 (24.2) | 67 (35.8) |
| Visual deficit | 541 (33.9) | 430 (32.2) | 77 (31.7) | 55 (29.4) |
| Hearing deficit | **173 (10.8)*** | **218 (16.3)*** | 25 (10.3) | 26 (13.9) |
| Behavioral Changes | 366 (22.9) | 383 (28.7) | 39 (16.0) | 38 (20.3) |
| Fatigue | **950 (59.5)*** | **860 (64.5)*** | **87 (35.8)*** | **104 (55.6)*** |

*Pearson’s Chi-square test with Yates correction, p < 0.01
